# The Impact of Physical Activity and Screen Time on Motor Creativity in Kindergarteners

**DOI:** 10.3390/children12020116

**Published:** 2025-01-22

**Authors:** Rafat Ghanamah

**Affiliations:** 1 Special Education Department Sakhnin College for Teacher Education, Sakhnin 3081000, Israel; rafatgan1980@sakhnin.ac.il; 2 Institut für Mathematische Bildung, University of Education Freiburg, Kunzenweg 21, 79117 Freiburg, Germany

**Keywords:** motor creativity, physical activity, screen time, cognitive functions, child development, early childhood education

## Abstract

Background/Objectives: Motor creativity and physical activity are essential to early childhood development, impacting physical, cognitive, and socio-emotional development. This study investigates the relationships among motor creativity, motor working memory (MSTM), screen time, and physical activity (PA) in kindergarten children, focusing on the mediating roles of cognitive functions and screen time. Methods: Data were collected from 124 Arab Israeli kindergarten children through assessments of Thinking Creatively in Action and Movement (TCAM) for motor creativity and the Hand Movement Test for MSTM. Parents reported the children’s screen time and days engaged in moderate-to-vigorous physical activity (MVPA). Results: The results show significant positive associations between motor creativity and both MSTM and PA, underscoring the role of cognitive processes in creative motor expression. Linear regression and mediation analyses showed that MSTM significantly mediates the relationship between PA and motor creativity. Conversely, screen time negatively correlates with PA and motor creativity, serving as a significant mediator that restricts opportunities for physical and creative activities. Conclusions: This study emphasizes the bidirectional relationship between PA and motor creativity, wherein diverse physical activities stimulate creativity, and creative movements encourage active participation. The combined mediating effects of MSTM and screen time highlight the complexity of these relationships, suggesting the need for integrated interventions. The findings inform early childhood education by advocating for strategies that promote physical activity, enhance cognitive functions, and limit excessive screen time, fostering holistic development in young children.

## 1. Introduction

Motor creativity and physical activity are essential for early childhood development, positively impacting physical, cognitive, social, and emotional well-being [1,2,3]. They allow children to explore, make judgments, solve issues, and use symbolic thinking, all of which are necessary components of creativity [4,5,6]. Motor creativity is the capacity to produce original and flexible movement sequences, enabling individuals to think critically, problem-solve, and respond effectively to unpredictable circumstances [7]. Physical activity, especially moderate-to-vigorous physical activity (MVPA), supports children’s physical health, cognitive functions, and social skills, serving as a critical component of holistic development [8]. The interplay between these constructs, especially their bidirectional relationship in young children, remains understudied.

Motor creativity can encourage preschoolers to experiment with movement, leading to increased participation in diverse physical activities. Conversely, physical activity can stimulate motor creativity by providing opportunities for varied movement experiences [9,10]. A deeper understanding of this relationship can inform the development of early interventions designed to enhance both physical and creative competencies.

### 1.1. Theoretical Background

Motor creativity and physical activity are interrelated aspects of early childhood development, both contributing to and benefiting from children’s cognitive, physical, and socio-emotional growth.

Creativity, the ability to generate novel ideas, is increasingly recognized as a vital cognitive skill for young children [11,12]. Creativity includes cognitive processes such as flexibility, originality, and abstract thinking; these skills are necessary for efficient problem-solving and adaptive behavior [13,14,15]. Neurocognitive research on the brain mechanisms underlying creativity has linked specific areas, such as the prefrontal cortex, anterior cingulate cortex, and default mode network, to creative thinking. These areas are involved with a variety of mental tasks, including ideation, cognitive flexibility, and problem-solving [16].

Creativity theories, including Guilford’s Structure of Intellect Model and Runco’s Theory of Creativity, suggest that divergent thinking and cognitive flexibility are critical components of creative problem-solving [15,17]. Given these theoretical frameworks, recent studies propose that creativity can serve a critical role in motor competence. The cognitive processes involved in creative thinking may impact a child’s acquisition and refinement of motor skills [18,19]. Creativity can appear in various expressions, including writing, problem-solving, drawing, and music composition [20,21]. Assessing creativity usually entails evaluating students’ outputs and work processes, particularly in assignments that emphasize internal cognitive processes and where progress may not be immediately apparent. This approach helps evaluate the efficiency of their creative thinking and problem-solving skills [22,23].

### 1.2. Physical Activity and Motor Development

Physical activity, specifically moderate-to-vigorous physical activity (MVPA), has been constantly linked to enhancing motor proficiency and overall health in children. The correlation between MVPA and motor capabilities is bidirectional: children with higher motor competencies are more likely to engage in physical activity, which enhances and upgrades their motor abilities [8,24]. This interaction is especially critical during early childhood (ages 1–7), which is characterized by rapid neurodevelopment and physical growth.

Clarke and Metcalfe’s [25] mountain of motor development metaphor affords a practical framework for understanding this process. They describe motor development as a progressive climb, where children in the early years gain central movement skills—such as running, jumping, and throwing—that serve as building blocks for more progressive and specialized skills essential in later sports and physical activities. These fundamental movement abilities outline the “foundation” necessary for ultimate physical activity, highlighting the importance of providing rich opportunities for practice and skill development during this stage.

The current literature supports this view, highlighting that creating enriched environments for structured and unstructured physical activities is essential for developing motor competence and physical literacy [26,27]. Motor competence refers to a child’s ability to execute a range of motor skills with proficiency and control, while physical literacy incorporates the motivation, confidence, and knowledge needed to engage in various physical activities across the lifespan.

The research proposes that motor creativity may act as a bridge between physical activity and motor development. Children with higher motor creativity tend to explore various and new movement patterns, developing their motor skills and increasing their involvement in physical activity [28,29]. By engaging in both structured and unstructured activities, children can not only refine their motor skills but also develop creative movement strategies, creating a positive feedback loop that reinforces their motor competence and physical literacy [3,30].

### 1.3. The Role of Cognitive Functions

Motor creativity is strongly connected to cognitive functions like motor working memory and executive function [31,32,33,34]. These cognitive abilities support the planning and execution of movements, which are essential for creative motor expression [31]. Research suggests that Motor memory contributes to the maintenance of attention and the integration of sensory feedback during complex motor tasks, potentially facilitating motor creativity through iterative learning processes [35,36,37].

### 1.4. Screen Time and Physical Activity

In modern contexts, increased screen time has become a barrier to physical activity and motor creativity. Excessive screen exposure has been related to decreased MVPA and reduced opportunities for creative movement exploration [38,39]. Nonetheless, not all screen time is disadvantageous; interactive media that fosters physical engagement, such as motion-sensing games, may offer unique opportunities to advance physical activity as well as motor creativity [40].

### 1.5. The Importance of MVPA Days

The incidence of engaging in MVPA (“MVPA days”) is another significant factor affecting motor creativity and physical development. Regular engagement in physical activity promotes an environment where children can steadily practice and upgrade their motor skills, thereby improving their competence for creative expression [9]. Evidence suggests that children who meet physical activity guidelines are more likely to develop advanced motor skills and demonstrate greater cognitive and creative capacities compared to their less active peers [9,41].

### 1.6. The Present Study

This study examines the relationships among motor creativity, motor working memory, screen time, and days of participation in moderate-to-vigorous physical activity (MVPA) among kindergarten children. While research has emphasized the distinct significance of motor creativity, working memory, and physical activity in child development, there remains a gap in understanding their combined influence and interactions within the same framework. Additionally, the role of screen time as a potential moderating or mediating factor in these relationships is underexplored.

Motor creativity is hypothesized to advance the physical activity levels of children by enabling diverse and innovative engagement in movement tasks. At the same time, motor working memory may support this creativity by aiding in the planning, execution, and adaptation of motor behaviors. However, screen time, as a primarily sedentary behavior, may diminish opportunities for physical activity, potentially restricting the enhancement of motor creativity and working memory.

### 1.7. Research Questions

The research seeks to answer the following questions:

- How is motor creativity associated with motor short-term memory in kindergarten children?
- What is the association between motor creativity and the number of days children engage in MVPA?
- How does screen time relate to motor creativity, motor short-term memory, and MVPA days?
- Do motor working memory and screen time mediate the association between motor creativity and MVPA days?

### 1.8. Objectives

The investigation seeks to achieve the following objectives:

- To test the direct relationships between motor creativity, motor working memory, screen time, and MVPA days.
- To determine whether motor short-term memory facilitates the association between motor creativity and physical activity patterns.
- To investigate the potential impact of screen time on the interplay among motor creativity, cognitive function, and physical activity.

The outcomes from this research will add to the expanding body of knowledge on early childhood development, offering insights into how cognitive, creative, and behavioral factors collectively shape children’s engagement in physical activity

## 2. Materials and Methods

### 2.1. Participants

One hundred twenty-four Arab Israeli kindergarteners participated in this study, including 64 females. The children were all between five and six years old, with a mean age of 68.47 months (±3.16 months). The children were Arabic speakers, talked in the same local dialect, and were enrolled from ten public kindergartens located in the northern area of Israel, representing middle–low socioeconomic status.

The current study was conducted after the approval of the research ethics committee of the Sakhnin Academic College for Teacher Education in Israel (11-2023-18A). Parental informed consent forms, issued by the Institutional Ethic Committee, were collected for children whose parents agreed to their participation in the study. In addition, prior to participation, assent was obtained from all children through an age-appropriate explanation of the study, ensuring their understanding and voluntary agreement. All procedures and methods complied with the guidelines of the Declaration of Helsinki and the human research procedure of the Local College for Teacher Education.

### 2.2. Measurements

#### 2.2.1. Cognitive Measure

Kaufman Hand Movement—Motor Short-Term Memory (MSTM) [42].

In this test, participants were required to mimic a random series of hand gestures, such as forming a fist, displaying the palm, or using the edge of their hand. The sequences differed in length, spanning from two to five movements. The test continued until the participant committed three errors in a row. The Cronbach’s alpha for this test was 0.89. In the current study, raw scores are reported.

#### 2.2.2. Creativity Measure

Torrance’s Thinking Creatively in Action and Movement (TCAM) Creativity Test [43].

This measure assesses preschool children’s creative thinking abilities as expressed through movement. The test, lasting approximately 10 to 20 min, was administered individually to children aged 3 to 8 years. It consisted of four activities:

1. How Many Ways: Children were asked to demonstrate different ways to walk or run.
2. Can You Move Like: Children showed how various objects or animals might move. This included six imaginative scenarios: four involved pretending to be an animal or object (e.g., a tree, rabbit, fish, or snake), while two involved roles linked to other contexts (e.g., driving a car or pushing an elephant off an object).
3. What Other Ways: Children were prompted to show alternative ways of placing a cup into a trash can.
4. What Might Be: Children were asked to demonstrate creative uses for a cup.

The first, third, and fourth activities were evaluated for fluency and originality, while the second activity was assessed for imagination. Fluency refers to the ability to generate multiple alternative movements. Originality is defined as the capacity to create novel, unique, or uncommon movements. Imagination captures the ability to empathize, fantasize, and adopt unusual roles.

Inter-rater reliability for the TCAM scoring was assessed among three trained research assistants. Cohen’s Kappa coefficients for fluency, originality, and imagination scores ranged from 0.86 to 0.92, indicating high levels of agreement.

Fluency was assessed by summing up the variety of responses recorded on the score sheets. Originality was determined using the measure’s norms, based on the most common responses from 500 children aged three to seven years. Imagination was evaluated using a five-point scale rating system (from 1 = no movement to 5 = excellent imitation).

The Torrance Tests of Creative Thinking (TTCT), including the TCAM, have demonstrated good reliability and validity in numerous studies (e.g., Torrance, 1981, [43]). The TCAM has shown good concurrent and predictive validity in relation to other measures of creativity.

#### 2.2.3. Physical Activity Measure

We asked the question, “On average, how many days a week does your son or daughter participate in moderate to vigorous physical activity for at least 60 min?” This measurement served as the foundation for assessing physical activity (PA). It has been shown to possess strong validity and reliability [44] and has been used widely in recent research [45,46,47,48]. The answer options were provided in 1-day increments, ranging from 0 to 7 days per week.

#### 2.2.4. Screen Time Measure

Screen time (ST) was assessed by asking the parents, “How many hours does your child spend on screens (smartphone, television, tablet, PC, etc.) on a regular weekday?”. The same question was also asked regarding a regular weekend. It is important to note that parents were clearly told to exclude time spent on electronic devices used for kindergarten activities.

The World Health Organization (WHO) recommends that children and adolescents aged 5 to 17 years participate in at least 60 min of moderate-to-vigorous physical activity each day. Additionally, they should spend no more than 120 min on leisure screen time and aim for 8 to 11 h of good-quality sleep each night.

### 2.3. Procedure

The children’s PA and ST scores were provided by their parents via an online questionnaire and a total of 124 parents completed the questionnaire.

Following the parents’ ratings, the children were tested individually in two sessions. All assessments were conducted in the morning hours (before noon) to ensure consistency in cognitive functioning. The children were evaluated in the first session on cognitive functioning through the Motor Short-Term Memory (MSTM) test. Then, ten minutes later, the second session took place, during which the children’s creativity was assessed using the TCAM test procedure. All testing was conducted individually in a quiet room, ensuring ample space for each child to move freely. The assessments took place during the second trimester of the school year (February to March) and were managed by three instructed assessors.

### 2.4. Data Analysis

Data analysis was performed using a combination of descriptive and inferential statistics. Firstly, descriptive statistics were calculated for all variables, including means and standard deviations. To test the associations between motor creativity, physical activity, screen time, and motor short-term memory, Pearson correlation coefficients were calculated.

Mediation analyses were implemented to measure the mediating role of screen time and motor short-term memory in the association between physical activity and creativity. The Sobel test was used to determine the significance of the indirect effect of physical activity on creativity through motor short-term memory and screen time. This test assesses whether the mediation effect is statistically significant by calculating the z-value and *p*-value for the mediated pathway.

To measure the mediating role of motor short-term memory and screen time in the association between physical activity and creativity, a mediation analysis was implemented. The Sobel test was used to determine the significance of the indirect effect of physical activity on creativity through motor short-term memory and screen time separately and combined. This test assesses whether the mediation effect is statistically significant by calculating the z-value and *p*-value for the mediated pathway.

Specifically, the analysis followed these steps:

Regression Analysis. We conducted three regression analyses:

The first regression examined the effect of physical activity on motor short-term memory and screen time.

The second regression assessed the impact of motor short-term memory and screen time on creativity, controlling for physical activity. The third regression analyzed the direct effect of physical activity on motor creativity.

Sobel Test. The Sobel test was applied to evaluate the significance of the mediation effect. The formula used for the Sobel test was *z*-value = *a* × *b*/SQRT (*b*^2^ × *s*_a_^2^ + *a*^2^ × *s*_b_^2^), where *a* is the regression coefficient for the path from physical activity to motor short-term memory and screen time, *b* is the regression coefficient for the path from motor short-term memory and screen time to creativity, and *s*_a_ and *s*_b_ are the standard errors of these coefficients.

All analyses were conducted using IBM SPSS Statistics Data Editor (version 30), with a significance level set at *p* < 0.05.

## 3. Results

Table 1 provides the children’s data regarding age and gender and the results of participants on the motor short-term memory test, screen time, and physical activity measures (as reported by the parents).

Table 2 presents the kindergarteners’ standard scores and standard deviations on the TCAM variables.

### 3.1. Correlations Between the Motor Short-Term Memory, Screen Time, Physical Activity, and TCAM (Creativity) Measures

Pearson correlation coefficients reveal significant associations between creativity measures and all other measures. Additionally, a significant correlation was observed between physical activity and all other measures. Notably, screen time had a significant negative correlation with all variables (see Table 3).

### 3.2. The Mediation Role of the MSTM in the Association Between Physical Activity and Motor Creativity

Linear regression analyses revealed significant associations between total physical activity and the MSTM (cognitive function) task (*F* (1, 122) = 53.81, *p* < 0.001) and between the MSTM task and total creativity (*F* (1, 122) = 54.05, *p* < 0.001). Additionally, physical activity was found to significantly predict total creativity (*F* (1, 122) = 57, *p* < 0.001).

To examine the mediating role of MSTM in the relationship between physical activity and motor creativity, a Sobel test was conducted. The obtained values were *a* = 0.39, *s*_a_ = 0.05, *b* = 2.92, and *s*_b_ = 0.71. The calculated z-value of approximately 3.60 indicates a significant mediation effect (*p* < 0.001), with a standard error of approximately 0.31. These results suggest a highly significant mediating role of MSTM in the association between playfulness and creativity among preschool children.

### 3.3. The Mediation Role of Screen Time in the Association Between Physical Activity and Motor Creativity

Linear regression analyses revealed significant associations between total physical activity and screen time (*F* (1, 122) = 112.19, *p* < 0.001) and between the screen time and total creativity (*F* (1, 122) = 106.24, *p* < 0.001). Additionally, physical activity was found to significantly predict total creativity (*F* (1, 122) = 57, *p* < 0.001).

To examine the mediating role of screen time in the relationship between physical activity and motor creativity, a Sobel test was conducted. The obtained values were *a* = −0.42, *s*_a_ = 0.04, *b* = −0.94, and *s*_b_ = 0.14. The calculated z-value of approximately 5.54 indicates a significant mediation effect (*p* < 0.001), with a standard error of approximately 0.07. These results suggest a highly significant mediating role of screen time in the association between playfulness and creativity among preschool children.

### 3.4. Combined Mediation Effects of Motor Working Memory and Screen Time on the Relationship Between MVPA and Motor Creativity

To test the mediation effect of motor working memory and screen time on the relationship between MVPA and motor creativity, a Sobel test was performed. The total indirect effect was calculated by summing the individual indirect effects through motor working memory and screen time. The total indirect effect was found to be 4.81, and the standard error for the combined indirect effect was 1.06. The Sobel test statistic was *z* = 4.53, which exceeds the critical value of 1.96, indicating that the combined indirect effect is statistically significant. Therefore, motor working memory and screen time significantly mediate the relationship between physical activity and motor creativity.

## 4. Discussion

The current study examined the complex associations between motor creativity, motor working memory (MSTM), screen time, and physical activity (PA) among kindergarten children, focusing on the mediating roles of cognitive functions and screen time. The results contribute valuable insights into the interconnections between these developmental factors and their effects on early childhood education and physical development.

### 4.1. Motor Creativity and Motor Working Memory (MSTM)

This study found significant associations between motor creativity and motor working memory, highlighting the fundamental role of cognitive functions in developing creative movement. This supports previous research suggesting that creative motor tasks, which demand flexible thinking and adaptation, rely on cognitive functions such as motor working memory [32,49,50]. In particular, the ability to retain and control information related to movement patterns enables the execution of more original and flexible motor responses [34]. These findings align with intervention strategies emphasizing integrated physical and cognitive tasks, such as obstacle courses, problem-solving movement games, and interactive physical education activities [3,34]. Programs that combine physical and cognitive challenges demonstrate the potential to enhance motor creativity and working memory simultaneously.

### 4.2. Physical Activity and Motor Creativity

One of the most striking findings of the present study was the significant correlation between physical activity (PA) and motor creativity. The outcomes denote that higher levels of MVPA were associated with greater motor creativity, supporting the bidirectional association between these variables. This aligns with earlier studies indicating that children who participate in diverse physical activities are more likely to develop motor skills that enhance creative expression [9,28,51]. Additionally, structured and unstructured physical activities provide opportunities for children to explore and experiment with diverse movement patterns, which is essential for the development of motor creativity [30].

Practical programs such as SPARK (Sports, Play, and Active Recreation for Kids) emphasize the value of integrating structured physical activities to boost motor creativity [52,53]. These programs encounter children with various movement patterns, promoting physical fitness as well as creative expression. Furthermore, initiatives such as Forest Schools foster outdoor activities that provide children the opportunity to explore and innovate through unstructured play, further supporting the link between PA and motor creativity [54].

### 4.3. Mediating Role of Motor Working Memory

The linear regression analyses and Sobel test revealed that motor working memory significantly mediates the relationship between physical activity and motor creativity. This suggests that the cognitive processes underlying working memory may support children’s ability to engage in creative motor tasks. The ability to store, maintain, and control information about physical movements allows children to adapt and innovate during physical play. These results align with research by Diamond [37] and Dietrich and Haider [36], which emphasizes the role of executive functions in complex motor tasks.

Programs like CogniFit and Move-to-Improve illustrate the possibility for incorporating working memory training with physical activities. These programs demonstrate that directing cognitive functions through movement-based tasks may enhance creativity as well as motor competence in young children [55]. By engaging children in missions that join memory challenges with physical activity, such interventions support whole development.

### 4.4. Mediating Role of Screen Time

One significant finding was the mediating role of screen time in the relationship between physical activity and motor creativity. The negative correlation between screen time and physical activity supports the existing literature that links excessive screen exposure to lower levels of physical activity [38]. Moreover, the notable mediating effect of screen time suggests that it may restrict opportunities for children to engage in physical activities, thus hindering their creative motor development. This aligns with research indicating that sedentary behaviors, such as excessive screen time, negatively impact both physical activity and cognitive development [39].

Not all screen time is detrimental, however. Interactive media, such as motion-sensing games, provide innovative ways to combine physical activity with creative movement (Lee-Cultura et al. [40]). Programs advancing balanced technology use, such as ActiveHealthyKids.org, propose strategies to reduce sedentary screen behaviors while promoting active play and motion-based digital engagement [56].

### 4.5. Combined Mediation Effect of Motor Working Memory and Screen Time

The Sobel test for the combined mediation effect of motor working memory and screen time further emphasized the complexity of the relationship between physical activity and motor creativity. The significant mediation effect suggests that both cognitive and behavioral factors play a crucial role in shaping children’s creative motor behaviors.

Inclusive methods, like SHAPE America’s Comprehensive School Physical Activity Program (CSPAP), refer to the interplay of physical activity, cognitive skills, and screen time. These programs aim to create balanced opportunities for physical activity while supporting cognitive engagement and minimizing sedentary behaviors. By integrating strategies and methods to boost motor working memory and control screen time, such interventions offer a practical framework for advancing motor creativity in young children.

### 4.6. Implications for Early Childhood Education and Intervention

The findings emphasize the importance of enhancing physical activity to foster both motor creativity and cognitive development in early childhood education and intervention programs.

Both educators and parents should encourage children to engage in sufficient daily physical activity, as our findings suggest a positive association between physical activity and motor creativity. Future research could explore whether specific types of physical activity (e.g., structured versus unstructured) yield distinct benefits for motor creativity and cognitive development. Additionally, since motor working memory plays a crucial role in enhancing motor creativity, exercises designed to target working memory can be included in physical activity routines to further promote creative motor behaviors.

The detrimental effect of excessive screen time on physical activity and creativity highlights the importance of limiting sedentary behaviors in early childhood environments. Strategies could include promoting active play, encouraging outdoor activities, and utilizing interactive media that fosters movement. Integrating these approaches into early childhood education and intervention programs may enhance both physical and cognitive development, laying a strong foundation for lifelong motor and creative skills.

## 5. Conclusions

This study sheds light on the intricate relationships between motor creativity, motor working memory, screen time, and physical activity in young children, providing valuable insights for early childhood education and intervention programs. The findings underscore the critical role of physical activity in fostering motor creativity and highlight the mediating effects of motor working memory and screen time in these relationships. By emphasizing the importance of promoting physical activity, supporting cognitive development, and managing screen time in early childhood settings, the study contributes to a growing understanding of how creativity and physical activity interact to shape children’s overall development.

These findings align with established theories of motor development, such as Clark and Metcalfe’s “mountain of motor development” metaphor, which emphasizes the foundational role of early motor skill acquisition in building lifelong physical activity and motor proficiency. According to this framework, the early childhood years are crucial for developing the fundamental movement skills that serve as building blocks for more advanced motor and creative abilities. Additionally, the concept of physical literacy highlights the need for diverse opportunities to practice and refine motor skills, as well as the importance of fostering confidence and motivation in physical activity engagement.

However, while the findings are significant, several limitations should be noted. The cross-sectional design restricts causal inferences, and future research using longitudinal designs could explore the directionality and long-term impacts of these relationships. Additionally, the reliance on parent-reported data for physical activity and screen time introduces potential biases; future studies should integrate objective measures, such as accelerometers, to enhance data accuracy. Furthermore, the focus on motor working memory and screen time as mediators limits the scope of the cognitive factors examined. Future research could investigate other cognitive functions, such as attention and inhibition, to provide a more comprehensive understanding of the mechanisms underlying motor creativity. Finally, distinguishing between different types of screen time—such as educational versus entertainment—may uncover nuanced effects on motor creativity and physical activity.

Despite these limitations, the study’s contributions are notable. The findings support the development of targeted interventions that integrate physical activity and cognitive challenges to enhance motor creativity. Programs designed to reduce sedentary behaviors and encourage diverse, structured, and unstructured physical activities hold promise for fostering both creative and physical development in early childhood. Grounded in theories of motor development, these interventions could capitalize on the critical periods of early childhood to establish foundational skills that support lifelong physical and creative competencies. As research in this field progresses, future studies addressing the outlined limitations will further elucidate the complex interplay between cognitive, physical, and creative domains, paving the way for more effective strategies to support children’s holistic development.

## Figures and Tables

**Table 1:** Age and gender data, and the participants’ motor short-term memory, screen time, and physical activity results.

Variable/Measure	M (SD)
Age (months)	67.57 (3.16)
Gender (females)	64
Motor Short-Term Memory	8.38 (1.08)
Screen Time (hours a day)	1.88 (0.94)
Physical Activity (days a week)	3.71 (0.82)

**Table 2:** Means and standard deviations on the TCAM measures.

Measure	M (SD)
TCAM Fluency	97.17 (10.65)
TCAM Originality	95.68 (8.86)
TCAM Imagination	92.98 (8.41)
TCAM Total Score	95.28 (9.02)

TCAM = Torrance’s Thinking Creatively in Action and Movement.

**Table 3:** Pearson correlations between motor short-term memory, screen time, physical activity, and TCAM (creativity) measures.

Variable/Measure	MSTM	ST	PA	FL	OR	IM	TCR
MSTM	1	−0.57 **	0.58 **	0.54 **	0.50 **	0.58 **	0.56 **
ST		1	−0.69 **	−0.64 **	−0.66 **	−0.68 **	−0.68 **
PA			1	0.53	0.53	0.60 **	0.57 **
FL				1	0.94 **	0.89 **	0.97 **
OR					1	0.89 **	0.97 **
IM						1	0.95 **

Note. MSTM = motor short-term memory measure; ST = screen time; PA = physical activity; FL = fluency; OR = originality; IM = imagination; TCR = total creativity measure. ** *p <* 0.001.

## Data Availability

The dataset is available on request from the author.
